# The role of personality and experience in bimodal signal integration in a territorial poison frog

**DOI:** 10.1186/s12983-026-00624-3

**Published:** 2026-07-18

**Authors:** Dhanya Bharath, Mélissa Peignier, Phin Anibal, Noëlle Tschirren, Max Ringler, Eva Ringler

**Affiliations:** 1 Division Behavioural Ecology, Institute of Ecology and Evolution, University of Bern, Bern, Switzerland; 2 Ludwig Maximilian University of Munich, Munich, Germany; 3 Messerli Research Institute, University of Veterinary Medicine Vienna, Vienna, Austria; 4 Institute of Electronic Music and Acoustics, University of Music and Performing Arts Graz, Graz, Austria; 5 Department of Behavioral & Cognitive Biology, University of Vienna, Vienna, Austria; 6 Department of Evolutionary Biology, University of Vienna, Vienna, Austria

**Keywords:** Multimodal signals, Aggressiveness, Territoriality, Poison frogs, Anurans

## Abstract

Multimodal communication, in which multiple signal modalities are combined to produce a single compound signal, is employed by many species and can mediate a wide range of fitness-relevant behaviours. While the form and function of multimodal signals have been extensively studied, we know little about how individual traits such as personality and experience influence receivers’ responses to these signals. We investigated how aggressiveness and experience affect the behavioural responses to bimodal signals in the brilliant-thighed poison frog *Allobates femoralis*. In this species, male advertisement calls constitute a fixed bimodal signal, as the production of sound is coupled with the inflation of the vocal sac, which adds a visual cue. We presented territorial males with a synthetic advertisement call (acoustic cue) and a model frog with a pulsating vocal sac (visual cue), and manipulated signal ambiguity by varying the spatial distance between the two components. We predicted personality and experience would affect how males responded to the bimodal signal depending on its level of ambiguity, and expected younger and/or more aggressive males to respond faster and to attack the visual cue more frequently than older or less aggressive males, especially under high signal ambiguity. Contrary to our predictions, neither aggressiveness nor experience influenced response latency or attack likelihood. Instead, all males displayed a consistent population-level pattern: as the spatial separation between the visual and acoustic signal components increased, they were less likely to attack the model, and their initial and overall interactions shifted from the visual model to the sound source. These findings suggest that the fixed bimodal signals in *A. femoralis* selected for robust and uniform receiver responses to maintain reliable communication during high-stakes social interactions such as territorial defence. Aggressive receiver responses seem to be dual-staged, consisting of far-field phonotaxis to the acoustic signal component and visual and multimodal signals only becoming relevant during near-field aggression. Future studies should compare how ecological and social factors shape receivers’ responses across species with varying degrees of coupling between the signal components in order to better understand the evolution of multimodal signalling and of the diversity of receiver strategies.

**Supplementary Information:**

The online version contains supplementary material available at https://doi.org/10.1186/s12983-026-00624-3.

## Background

Multimodal communication is defined as communication via composite signals that are received through more than one sensory channel [1]. Such signals are widespread across the animal kingdom and occur in many fitness-relevant contexts, including mate recognition, courtship, aggression, and territorial defence [2–5]. Despite the importance of multimodal communication for survival and reproduction, previous studies have primarily focused on how, on average, receivers respond to multimodal signals [6–8]. However, to date, relatively little is known about the role of between-individual variation in perception and response to multimodal signals.

One potential source of such between-individual variation is animal personality, defined as consistent behavioural differences among individuals across time and/or contexts [9]. Personality traits may influence the response to multimodal signals [10]. For example, more aggressive individuals might respond to any signal, even if some components are missing or ambiguous, while less aggressive or shyer individuals may only react to unambiguous multimodal cues. Individual differences may also emerge in how receivers prioritise one sensory component over another. Another potential source of between-individual variation in reaction to multimodal signals may come from the experience individuals have with the signal [11]. Younger or less experienced individuals may struggle to interpret and respond appropriately to ambiguous or conflicting multimodal signals compared to older, more experienced individuals.

Bimodal signalling is a specific case of multimodal signalling involving the simultaneous use of two sensory modalities. It is particularly well-studied in anuran amphibians, where it plays a key role in courtship and territorial aggression [12–14]. In some contexts, the two stimuli are “free”, and one signal can be displayed without the presence of the other [6]. For example, in the foot-flagging frog *Staurois guttatus*, males combine advertisement calls with conspicuous foot movements [15–17]. In contrast, other species exhibit “fixed” bimodal signals, where the two components are mechanically coupled and cannot be separated. For instance, in many calling frogs, the acoustic signal is always accompanied by the inflation and deflation of the vocal sac, which provides a visual cue [18, 19].

Males of the brilliant-thighed poison frog, *Allobates femoralis* (Dendrobatidae sensu [20]), provide an excellent model for studying the effect of personality and experience on behavioural responses to bimodal signals, as both factors have been investigated in this species across different contexts. *A. femoralis* is a diurnal, ground-dwelling species that inhabits the lowland rainforests of the Amazon basin and the Guiana Shield. Males establish and defend territories throughout the breeding season using a fixed bimodal signal that combines prominent advertisement calls with the visual cue of a pulsating vocal sac [21]. While males use distinct close-encounter courtship calls when they suspect another frog in their territory to be a female, they feature no distinct close-encounter call for males [22]. Possessing a territory is essential for *A. femoralis* males to obtain mating partners [23], prompting them to aggressively defend their area against intruders [13, 21, 24, 25]. Effective defence therefore requires males to accurately perceive and react to bimodal signals emitted by conspecific rivals. Territorial interactions in *A. femoralis* typically involve a behavioural sequence of calling, orientation, approach, and attack, representing an escalation in response intensity. While the first three stages of the response can be reliably elicited through acoustic playback of conspecific calls alone [13, 21, 26], physical attacks on intruders occur only when both the acoustic and visual components are presented concurrently [13, 24, 27]. These attacks are consistently performed as long as visual and acoustic cues are presented within 12 cm spatial separation, but decrease sharply when the two cues are separated by 25 cm or more, indicating that increasing spatial separation disrupts signal integration [27]. Previous research suggests that experience modulates how males respond to bimodal signals, as younger individuals are more likely than older males to attack a non-threatening model (e.g., a frog model without a vocal sac) when it is presented alongside a male call [28]. In addition, *A. femoralis* males exhibit consistent personality traits, such as aggressiveness, which influences how quickly males approach an acoustic playback of conspecific calls placed within their territory [29, 30]. Hence, both experience and personality are known to shape territory defence behaviour in *A. femoralis*.

The aim of this study was to investigate how the personality trait aggressiveness and experience affect behavioural responses to bimodal signals in *A. femoralis* males. We presented territorial males with a synthetic playback of advertisement calls (acoustic component) and a model frog with a pulsating vocal sac (visual component), varying the spatial distance between the two stimuli to manipulate signal ambiguity. We predicted that younger and/or more aggressive males (with less experiences and/or inhibitory control) would respond more quickly and attack more frequently than older and/or less aggressive males, regardless of the spatial separation between the stimuli. Conversely, we expected older and/or less aggressive males (with more experience and/or inhibitory control) to adjust their responses to the spatial separation, showing slower reactions and fewer attacks with increasing distance. Finally, we predicted that younger and/or more aggressive males would approach the model frog first, even when the two signal components were widely separated.

## Methods

### Study site and procedure

We carried out fieldwork in the Nouragues Nature Reserve in French Guiana (4°02′ N, 52°41′W), near the Pararé field camp of the CNRS Nouragues Ecological Research Station [31]. We conducted experiments during the breeding season in April and May 2023 and used two local populations of *A. femoralis*, a natural population on the mainland near the field camp, and an experimental population introduced on an island in front of the field camp [32, 33]. We conducted our experiments every day during the frog’s most active hours between 1000 and 1800 h [21].

For our experiments (see sections “Bimodal test” and “Personality test”), we localised calling males in the study area. *Allobates femoralis* males have central perch territories, with an average diameter of the central area of 3.7 m and an average diameter of the defended area of 13.9 m [34], thus testing only calling males ensured that our test setups, with a testing distance of 2 m, were always placed within the territory of the focal male. We tested each individual only once per day in either a bimodal or a personality test. We randomised the order of the bimodal and personality tests for each individual to avoid any effects of the experimental sequence. After each test, we caught the focal frog using a transparent plastic bag, to minimise stress and direct contact, and photographed each individual on their dorsal and ventral sides against a reference scale. This procedure allowed us to visually identify each individual using their distinct ventral pattern directly in the field, and later confirm the identification using the pattern-matching software Wild-ID [35]. We later measured the snout-urostyle length from dorsal images using the software Image J [36] and used it as a proxy for age, based on the rationale that amphibians have continuous growth throughout life, even after sexual maturity [37].

### Bimodal test

**Fig. 1:**
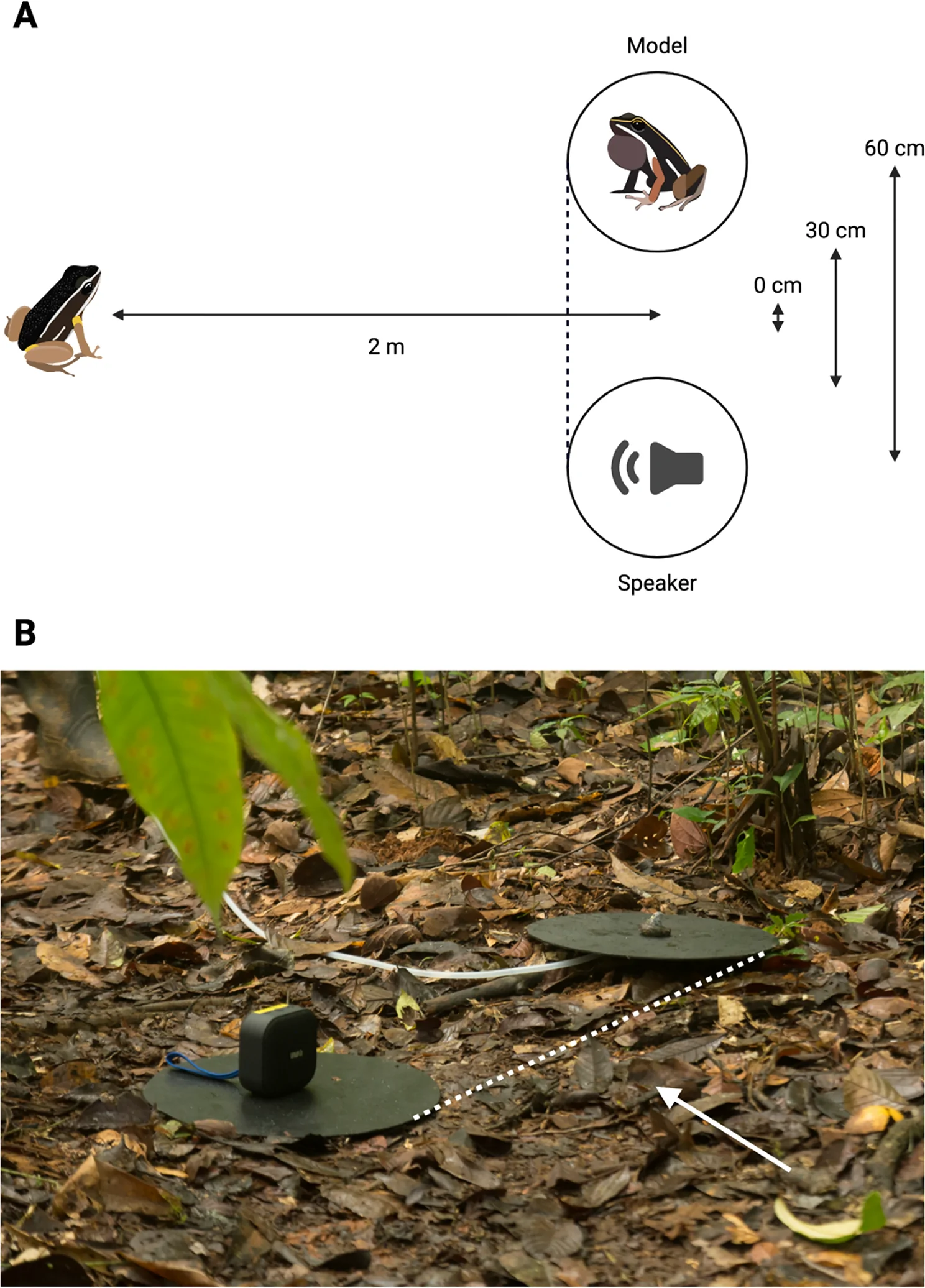
(**A**) Experimental setup for the bimodal test. The model frog (visual cue) and the speaker (acoustic cue) are placed 2 m in front of the focal individual. The three different treatment conditions include the model and the speaker placed together (0 cm apart), 30 cm apart and 60 cm apart. The dashed line represents the imaginary tangent between the discs to cross for the experimenter to declare that the focal frog reached the experimental setup. (**B**) Experimental setup of the bimodal test. The dashed line indicates the imaginary tangent between the discs to reach the experimental setup, and the arrow indicates the approach direction of the focal frog

We investigated the responses of individual males to bimodal signals in a territory defence context. We presented calling males with a model frog with a pulsating vocal sac and an acoustic playback of advertisement calls. We made the model frogs using a hand-sculpted clay form cast with latex (Esprit Composite, Paris, France) to result in moulds that we then filled with figure casting compound (Artestone, CREATEX trend-design GmbH, Lindenberg, Germany). We created the vocal sacs using silicone (Ecoflex 00–50 or Ecoflex 00–10, Smooth-On Inc., Macungie, PA, USA) (Figs. 1). We positioned the model frog in a sitting position on top of a spike that allowed us to stick it to the ground. We fixed a two meter long silicone tube to the underside of the frog and connected it to a blower bulb to inflate and deflate the vocal sacs (Figs. 1). The pulsation of the vocal sac was manually operated in synchrony with the standardised, synthetic advertisement calls, presented via a loudspeaker with an integrated audio player (A1, Mifa, HK Electronic Co., Hong Kong, P.R.C.). The synthetic stimulus featured the average spectral and temporal parameters of a nearby population and consisted of bouts of ten calls interspersed with equal periods of silence [38, 39]. The playback lasted for five minutes and was presented from a WAV file (16-bit, 44.1 kHz) with the speaker adjusted to produce the call with an SPL of at least 69 dB at the location of the frog. Each playback started with a 120 s period of silence to allow the frog to resume normal behaviour after our approach and setup.

Two experimenters were required to perform this experiment. We first localised calling males and placed the experimental setup at a distance of 2 m, using a laser rangefinder (DLE 50, Bosch, Stuttgart, Germany). We placed the model frog on top of a black PVC disc (30 cm diameter) and varied the ambiguity of the signal by varying the distance between the model frog and the loudspeaker. To do so, we placed the loudspeaker either right behind the model frog on the same black PVC disc (treatment 0), or on another black PVC disc located 30 cm (treatment 30) or 60 cm (treatment 60) next to the model frog. We randomized the speaker’s position relative to the model frog (left/right) within individuals. Once we had placed the experimental setup, both experimenters stood immobile 2 m behind the setup and started the playback. One experimenter handled the air pump to pulsate the vocal sac in synchrony with the speaker playback [8, 13], while the other experimenter recorded the behaviours of the frog using a digital voice recorder (WS-853, Olympus, Sony, Tokyo, Japan).

The experimenter recorded the first head-body orientation, first jump, first arrival at the experimental setup, defined as crossing an imaginary tangent touching both discs (dashed line in Fig. 1A), number of visits to the model or to the speaker (i.e., when the focal frog touched the model or speaker disc with at least one part of its body), whether the focal frog attacked the model [1] or not (0) and which element was visited first (model or speaker). We stopped each trial after five minutes when the playback ended. After each test, we measured the received sound pressure level (in dB) of the signal at the initial location of the frog using a sound pressure meter (SL-100, Voltcraft, Hirschau, Germany) to account for local variation due to vegetation density. In all cases, the received SPL was above the threshold known to elicit a phonotactic approach (i.e., 69 dB; [25]). If a male started calling continuously during the test, we stopped the experiment, as this is likely a sign that we positioned the setup outside its territory [34]. In such rare cases (< 5% of all trials), we tried to move the setup once. If the frog started calling again, we stopped for the day and tried again the next day if it was vocally active. Each individual was tested once per separation distance in the bimodal tests.

We blinded the names of the observation recording files prior to analysis and coding to avoid observer bias. From the recordings, we extracted the latency to reach the setup (in seconds), the number of visits made to the speaker and the model frog (at 30–60 cm separation), and the attacks to the model frog (“yes” or “no”). In trials where the male did not reach the setup (*N* = 23), we assigned a censored latency value of 300 s, corresponding to the total duration of the experiment. We also determined whether males visited the model frog or the speaker more frequently in treatments 30 and 60 by calculating the ratio:

$$\:\mathrm{l}\mathrm{o}\mathrm{g}\left(\frac{number\:of\:visits\:to\:the\:model\:frog+1}{number\:of\:visits\:to\:the\:speaker+1}\right).$$

### Personality test

To understand how the personality trait aggressiveness influences the response to bimodal signals, we used a standardised territorial defence test previously established for the same study species [29, 30]. We spotted calling males and placed the loudspeaker on a black PVC disc (30 cm diameter) at a distance of 2 m from the individual (measured using a laser rangefinder). We randomly presented playbacks of one of seven synthetic calls to the focal frog [30], all of which were different from the synthetic call used in the bimodal test. The playbacks varied in the lengths of their inter-note and inter-call intervals, which were chosen ad libitum within the natural range of a nearby population [38]. This was done to avoid habituation, as we tested the frogs multiple times in order to measure repeatability of the behaviours. The playbacks were presented from WAV files (16-bit, 44.1 kHz) and lasted five minutes, with a 30 s acclimation time before the first call started. The experimenter remained approximately one meter behind the loudspeaker and recorded the first head-body orientation, the first jump, and the arrival to the setup (i.e., when the frog touched the disc with at least one part of its body). Similar to the bimodal test, the personality test was suspended if the frog started calling during the test, and we assigned a censored latency value of 300 s to frogs who did not reach the PVC disk (*N* = 32). We moved the setup once and stopped for the day if the frog started calling again. The recorded files were transcribed following the same protocol as for the bimodal test.

### Statistical analysis

We conducted all statistical analyses in R v4.5.2 [40]. First, we confirmed that *A. femoralis* males exhibit consistent between-individual variation in aggressiveness-related behaviours by assessing the repeatability (R) of all the behaviours we measured during the personality test using the “rpt” function in the rptR package [41]. To achieve normal distribution, we log-transformed the latency until the initial head-body orientation and until the first jump, as well as the latency to reach the setup. We estimated repeatability from models fitted with a Gaussian error distribution. For all models, frog ID was included as a random effect, and bootstrapping and permutations were set to 1’000. We could not include the site (island vs. mainland) as a fixed effect to assess adjusted repeatabilities because of insufficient sample sizes, which did not allow models to converge.

We then investigated how the aggressiveness level obtained from the personality test, the body size and the treatment (i.e., 0, 30–60 cm between the two stimuli) influenced the following behavioural responses measured in the bimodal test: the latency to reach the setup, the likelihood to attack the model frog (binomial response, “yes” vs. “no”), the first visit (binomial response, “model” or “speaker”) and the ratio of the number of visits to the model over the number of visits to the speaker. The model with the latency to reach the setup used the full dataset while the model with the likelihood to attack the model frog used a dataset comprising only the data of males who reached the setup. Likewise, models with the first visit and the ratio of the number of visits to the model over the number of visits to the speaker both used a dataset comprising only the data of males who reached the setup in treatments 30 cm and 60 cm.

First, we removed one particularly small individual with an outlier body size (i.e. more than 3*SD below the mean) from our analysis. We ensured that there were no significant size differences in our two populations using an independent sample t-test (t = -0.11, *p* = 0.915). We also checked if we needed to include the repetition order and/or the relative location of the speaker (left vs. right of the model frog) in the models using Kruskal-Wallis or Wilcoxon rank sum tests when the data were not normally distributed. Neither the repetition nor the side influenced any of the variables (Additional file, Tables S1 and S2), and consequently, these variables were not included in further analyses.

In order to obtain the aggressiveness level of each individual, we extracted the best linear unbiased predictors (BLUPs) from random intercept models with the latency to jump in the personality test as the response variable and individual identity as a random effect. We used the latency to jump as a proxy for aggressiveness based on results from the repeatability analysis and based on results from previous studies [30, 42]. More aggressive individuals will have shorter latencies (i.e. react quicker to a potential competitor) compared to less aggressive males. We scaled the BLUPs by subtracting the mean and dividing by the standard deviation, and refer to these values as “aggressiveness” hereafter.

Finally, to investigate how aggressiveness, body size, and treatment influenced the behavioural responses measured in the bimodal test, we ran four Bayesian generalised linear mixed models (MCMCglmm package [43]), one for each response variable, with an interaction between aggressiveness score and treatment, and between body size and treatment as fixed effects. We also ran the same four models without the interaction to better interpret the results of the fixed effects. In all models, we included the individual identity nested within the “site” (i.e., island or mainland) as a random effect. We give specifications of the priors, number of iterations, burn-in and thinning interval in the additional file. We confirmed the absence of autocorrelation (correlation between lags < 0.1), sufficient mixing (via visual inspection of plots of MCMC chains), and that we ran the Markov chain for long enough (Heidelberg and Welch diagnostic tests [43]). Where we had significant results, we further investigated the results of the interactions using post hoc tests for the estimated marginal means (emmeans package [44]). We assumed statistical significance if the 95% credible intervals did not overlap zero.

## Results

In total, we conducted 104 personality trials and 93 bimodal trials with 31 males (15 island males and 16 mainland males). There was no significant difference in body size between island and mainland individuals (island: 2.91 ± 0.18 cm (mean ± SD); mainland: 2.92 ± 0.26 cm; t-test: t = -0.11, *p* = 0.915). We tested each frog on average three times in the personality test (range = 2–6), and we tested each frog once with each of the three treatments in the bimodal test.

In the personality tests, the latency to do a head-body orientation was not repeatable (*R* = 0.20, 95% CI [0–0.39], while both the latency to do a jump and the latency to reach the setup were significantly repeatable with respective values of *R* = 0.38, 95% CI [0.16–0.55] and *R* = 0.34, 95% CI [0.12–0.54]).

**Fig. 2:**
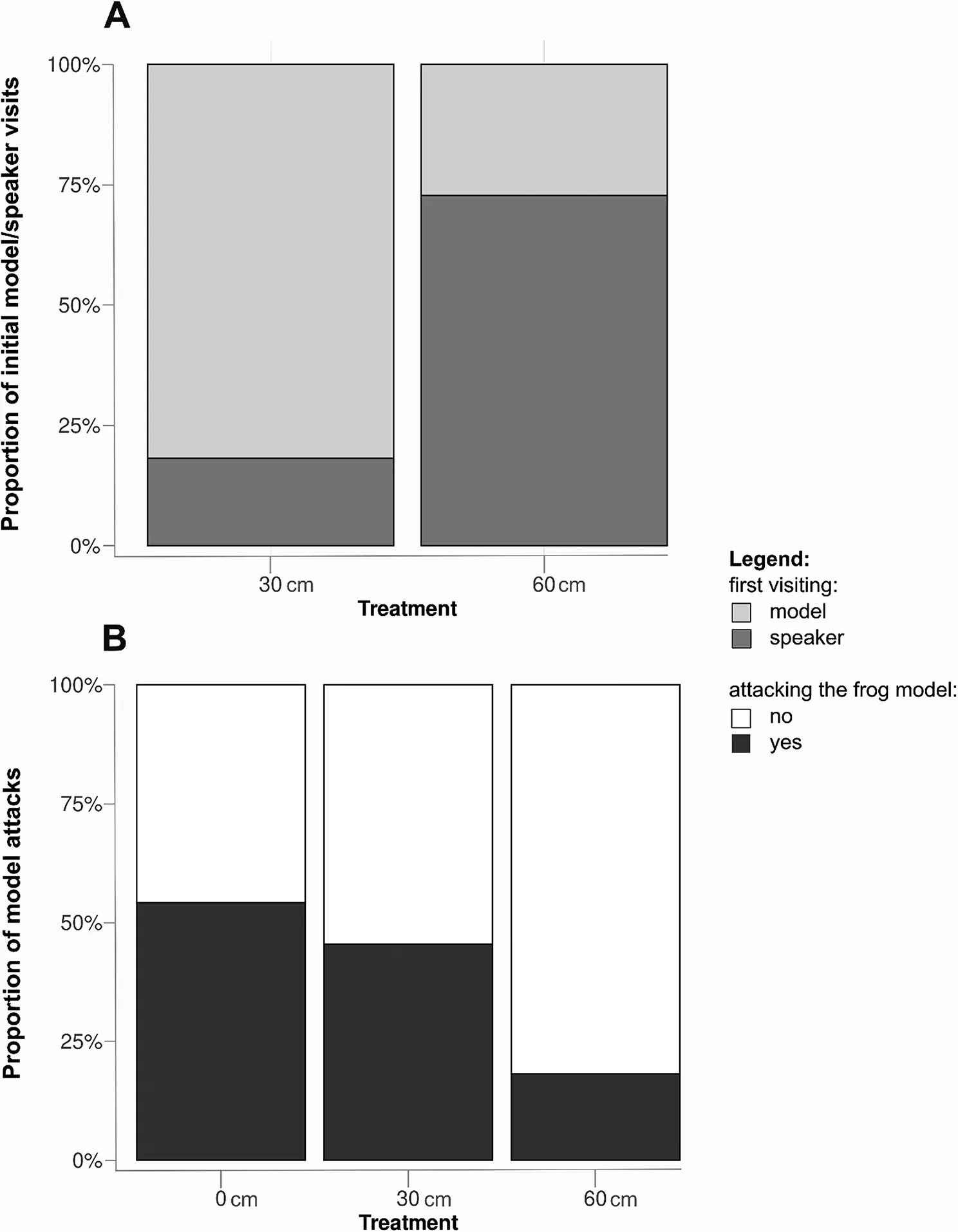
(**A**) Proportion of males who interacted first with the model frog and with the speaker when the spatial separation between the acoustic and visual cues was 30–60 cm. (**B**) Proportion of males who attacked the frog model (or not) when the spatial separation between the acoustic and visual cues was 0, 30–60 cm

In the bimodal tests, out of the 31 frogs tested, 24 frogs reached the setup in treatment 0 cm, and 23 frogs reached the setup in treatments 30 cm and 60 cm. Among the 23 frogs who reached the setup in treatment 30 cm, 20 frogs interacted with the model frog’s platform and 16 frogs interacted with the speaker’s platform. Likewise, among the 23 frogs who reached the setup in treatment 60 cm, 13 frogs attacked the model in treatment 0 cm, 10 frogs in treatment 30 cm, and five frogs in treatment 60 cm (Fig. 2B).

**Fig. 3:**
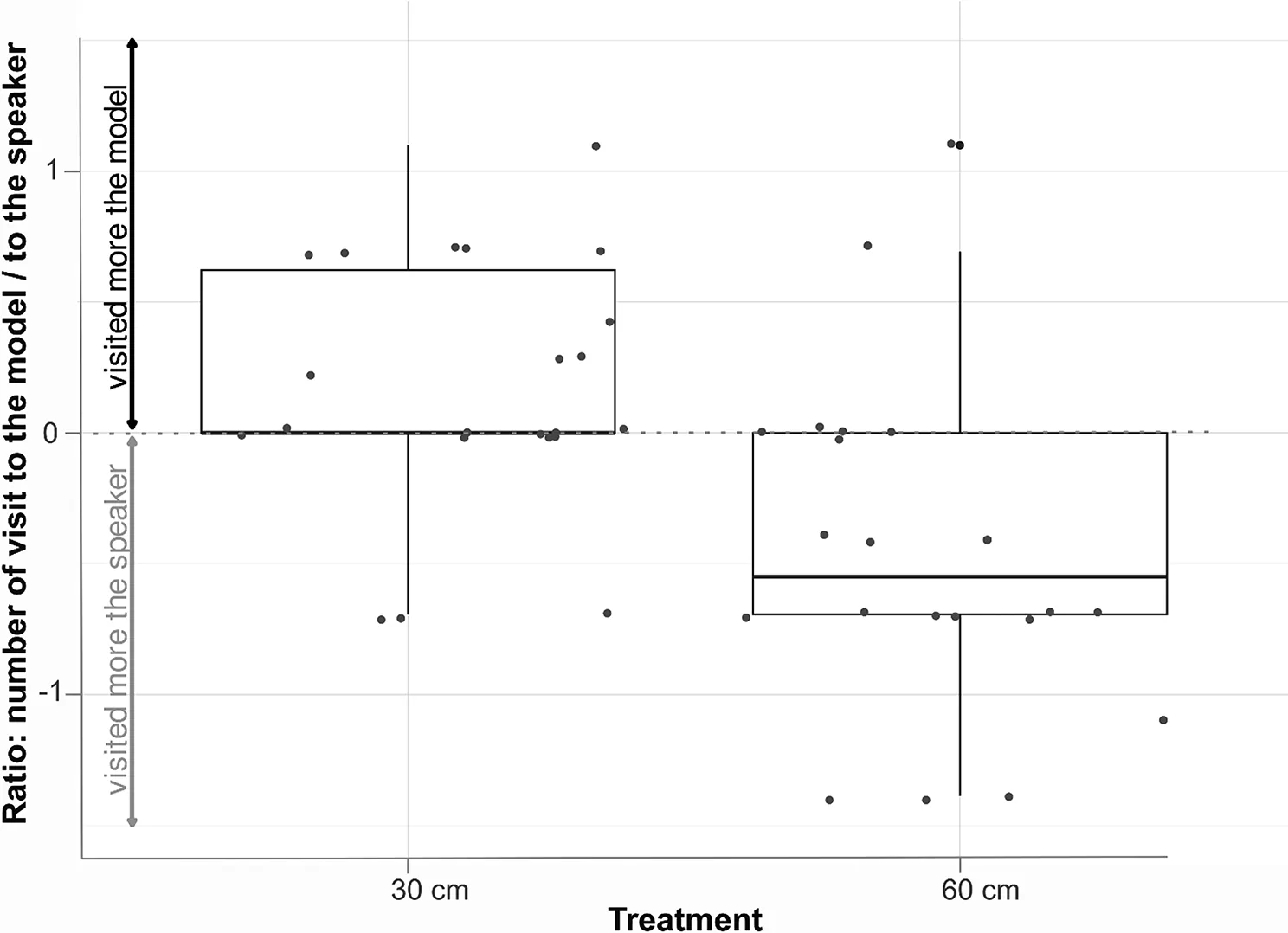
Log of the ratio of the number of visits to the model (plus 1) over the number of visits to the speaker (plus 1) in the bimodal test when the spatial separation between acoustic and visual cues was 30 or 60 cm. Ratio values below zero indicate that the male visited the speaker more often than the model. Ratio values over zero indicate the opposite. Box indicates the interquartile range. Bold horizontal lines indicate the median. Vertical lines extend to 1.5*the interquartile range. Dots indicate individual trials

We first investigated the influence of the interaction between treatment and aggressiveness, and between treatment and body size and none of these interactions influenced the behavioural variables measured during the bimodal test (Additional file, Table S3). We then had a closer look at the influence of each fixed effect independently and confirmed that the most aggressive males in the personality test were also the fastest males to reach the experimental setup in the bimodal test (Table 1). We also found that treatment influenced the likelihood to attack, with males being less likely to attack the model frog in treatment 60 cm compared to treatments 0 cm and 30 cm (Fig. 2B; pairwise contrast between treatment 0 cm and 60 cm: estimate = 2.61, 95%CI[0.59; 4.68] and between treatment 30 cm and 60 cm: estimate = 1.94, 95%CI[0.00; 3.84]). Likewise, treatment also influenced with which stimuli the first interaction happened and the ratio of the number of visits to the model to the number of visits to the speaker (Figs. 2A and 3). Males interacted first and more often with the model in treatment 30 cm, while they interacted first and more often with the speaker in treatment 60 cm (Table 1, *p* < 0.001 for the first interaction and *p* = 0.001 for the ratio).

**Table 1:** Parameter estimates and test statistics for the Bayesian linear mixed models, looking at the influence of treatment, aggressiveness, and body size on the behavioural variables measured during the bimodal test

*log(latency reach line)*	*post.mean*	*l-95%*	*u-95%*	*eff.samp*	*pMCMC*
location	125.5	0	5.37	9900	
ID	0.16	0.02	0.32	9520	
intercept	5.92	2.85	8.79	9900	**0.011**
treatment 30 cm	0.15	-0.11	0.42	9900	0.257
treatment 60 cm	0.1	-0.16	0.37	9900	0.466
aggressiveness	-0.31	-0.55	-0.08	8432	**0.018**
body size	-0.5	-1.38	0.35	9900	0.25
*likelihood to attack the model frog*	*post.mean*	*l-95%*	*u-95%*	*eff.samp*	*pMCMC*
location	10,596	0	42.65	6634	
ID	3.03	0	10.55	4550	
intercept	5.17	-11.18	18.97	6634	0.537
treatment 30 cm	-0.68	-2.37	1	6217	0.421
treatment 60 cm	-2.67	-4.79	-0.79	5223	**0.004**
aggressiveness	-0.61	-2.06	0.74	6634	0.33
body size	-1.22	-5.9	3.42	6341	0.552
*first interaction*	*post.mean*	*l-95%*	*u-95%*	*eff.samp*	*pMCMC*
location	2931	0	23.26	6634	
ID	0.48	0	2.28	5917	
intercept	1.41	-11.67	14.36	6634	0.852
treatment 60 cm	3.44	1.41	5.28	6371	**< 0.001**
aggressiveness	-0.18	-1.47	0.98	6996	0.772
body size	-1.08	-5.32	2.96	6634	0.599
*ratio visits to the model/visits to the speaker*	*post.mean*	*l-95%*	*u-95%*	*eff.samp*	*pMCMC*
location	115.3	0	3.72	9900	
ID	0.04	0	0.13	9900	
intercept	-0.57	-3.61	1.87	9900	0.556
treatment 60 cm	-0.64	-0.98	-0.32	9900	**0.001**
aggressiveness	-0.01	-0.28	0.25	9900	0.939
body size	0.31	-0.45	1.13	9900	0.434

Significant p-values (< 0.05) are written in bold

## Discussion

In the present study, we examined between-individual variation in how receivers respond to multimodal signals. We presented males of the neotropical poison frog *Allobates femoralis* with the species stereotypic, bimodal (acoustic and visual) signal, which is used by territory holders for advertisement, and by both contestants in territorial interactions. We varied the spatial distance between the two stimuli to manipulate signal ambiguity and investigated how a male’s body-size (i.e., a proxy for its age and level of experience) and personality shape its response to the signal. Contrary to our predictions, we found no evidence that either aggressiveness or experience influenced male responses to ambiguous bimodal signals, where the different sensory modalities are not clearly linked. Instead, males displayed a consistent population-level pattern: they were less likely to attack the model as the spatial separation between the visual and acoustic signal components increased. Male *A. femoralis* establish and defend territories annually during the reproductive season through advertisement calls [45], which function to repel rival males and attract females [46]. Possession of a territory is essential for obtaining mating partners [23], prompting males to aggressively defend their area against intruders by actively approaching them and engaging in physical fights [13, 21, 24, 25].

The absence of personality- and experience-based effects on responses to bimodal intruder signals in the present study contrasts with our expectations and with findings from other taxa, where personality traits often influence risk-taking and signal interpretation [47–49]. One possible explanation is that territorial interactions in *A. femoralis* might be under strong selective pressure, favouring stereotyped responses that minimise the risk of losing a territory, regardless of behavioural type. The high costs of territory loss could therefore select for a “better safe than sorry” strategy, where males attack any intruder even when ambiguity remains, independent of personality or experience. This interpretation is in line with the lack of a “dear-enemy” effect in this species, as territory holders showed similar phonotactic aggression to the calls of familiar and unfamiliar individuals [50–53].

Our results highlight that the effectiveness of multimodal signals depends more on their structural coherence than on the receiver’s individual traits. This finding supports theoretical models suggesting that fixed bimodal signals (i.e., where components are mechanically coupled) evolve under constraints promoting reliability and limiting interpretive flexibility [6, 54]. In contrast, free bimodal systems, where components can be expressed independently [15, 55–57], may contain more information, thus probably allowing for greater individual variation and personality-driven modulation of receiver responses. In *A. femoralis*, the fixed bimodal coupling of acoustic and visual components may promote more innate responses, reducing the influence of experience or learning. As a result, receiver responses to fixed multimodal signals are likely to show less variation between individuals than in free multimodal systems (see also [19, 58]).

Our results also show that males shifted their initial attention and overall interaction frequency from the sound source to the visual model as the spatial separation between components decreased across trials. It is likely that different sensory modalities are prioritised at different stages of the behavioural sequence (calling, orientation, approach and attack) typically observed during territorial interactions in *A. femoralis* and other poison frogs. For example, males in the chestnut-bellied flycatcher (*Monarcha castaneiventris*) assess different song and colour cues sequentially during conspecific recognition, a process which is influenced by habitat structure and variation in signal traits [55]. Similarly, male poison frogs may rely primarily on acoustic cues for initial detection and localisation of rivals, and then integrate visual information to confirm identity and trigger an attack once proximity is established [13, 46, 59–62].

Such subsequent prioritization of acoustic over visual signals is also in line with their physical propagation characteristics. Spherical spread and refraction around obstacles cause comparatively long-range propagation of acoustic signals in forests, whereas visual signals require a direct line of sight and are blocked much sooner, particularly on the forest floor [63]. In our experiment, when the spatial proximity between cues was large (60 cm), males seemingly interpreted them as separate stimuli and responded primarily to the acoustic signal rather than the visual one. Only at closer separations between the auditory and visual components (0–30 cm), males first approached and subsequently attacked the model and often remained there.

The increase in attack probability and the shift in focus from auditory to visual/bimodal cues with decreasing spatial separation align with previous findings that *A. femoralis* males require close spatial and temporal correspondence between signal components to perceive them as a single multimodal event, and initiate a full physical attack [24, 27]. Our results reinforce the idea that *A. femoralis* employs a fixed bimodal signalling system in which signal efficacy depends strongly on the physical coupling of its components.

However, there are also some methodological limitations that might have caused the absence of an effect in our study. On the one hand, our proxy for experience (i.e., body size) may not accurately capture age or prior territorial encounters. In a previous study on the same species, researchers presented the call of a ‘threatening’ intruder alongside a ‘non-threatening’ intruder (i.e., a robot frog mimicking a female) without any spatial separation between them. The authors had knowledge of the exact age (in years/generations) of the tested individuals and found that younger individuals were more likely to attack the non-threatening model than older ones. In our study, such data was only available for the island frogs, which is the reason why we had to fall back to size as a proxy for age. Their results therefore suggest that experience does indeed play a role in identifying and distinguishing rival individuals in a territorial context [28], and further indicate that our proxy for experience might not be sufficiently precise. Mark-recapture data or controlled rearing experiments could help disentangle the roles of ontogeny and learning in shaping multimodal signal processing. On the other hand, our measure of aggressiveness (i.e., the latency to jump in a territory defence test) might reflect the strength of the initial territory defence behaviour rather than decision-making during close-range interactions. Once an intruder is clearly identified, other personality traits, such as boldness, may become more relevant to attack decisions.

## Conclusions

In summary, male *A. femoralis* integrate bimodal territorial signals in a highly consistent manner, largely independent of aggressiveness or experience. These findings suggest that in species relying on fixed bimodal signals, selection favours robust and uniform receiver responses to maintain reliable communication during high-stakes social interactions such as territorial defence. Future studies should experimentally manipulate other forms of signal ambiguity – such as temporal asynchrony or conflicting information – to test whether personality or experience become relevant under more complex conditions. Comparative studies across dendrobatid species differing in the degree of coupling between signal components could further elucidate how ecological and social factors shape both the evolution of multimodal signalling and the diversity of receiver strategies. Finally, exploring how fluctuating ecological and social environments influence selection pressures on both signallers and receivers [64] will be essential for understanding the dynamic evolution of multimodal communication systems.

## Supplementary Information

Below is the link to the electronic supplementary material.

Supplementary Material 1

## Data Availability

The dataset supporting the conclusions of this article is available in the Open Science Framework repository, https://osf.io/d9rtx/overview?view_only=cbcad3a10fa64778b7d4e02c33970a42.
