# Supplementary material for: The role of personality and experience in bimodal signal integration in a territorial poison frog
Source: Front Zool. 2026 Jul 18;23:37. doi: 10.1186/s12983-026-00624-3 (PMC13673614; doi:10.1186/s12983-026-00624-3)
Supplement: Supplementary file 1 [file 12983_2026_624_MOESM1_ESM.pdf]

Additional file 1

Title: The role of personality and experience in bimodal signal integration in a territorial poison frog

Authors: Dhanya Bharath[1,2#], Mélissa Peignier[1#], Phin Anibal[3], Noëlle Tschirren[1], Max Ringler[1,4,5,6], Eva Ringler[1*]

Table S1: Results of Kruskal-Wallis rank sum tests investigating the influenced of the test repetition number on different variables.

Variable	*Kruskal-Wallis*	*p-value*
Log(latency reach line)	2.95	0.229
Number of visits to model frog	0.39	0.822
Number of visits to speaker	1.68	0.433
Attack to the model frog (yes/no)	1.62	0.445

Table S2: Results of Kruskal-Wallis and Wilcoxon rank sum tests investigating the influenced of the side on which the speaker was (left or right of the model) on different variables.

Variable	*Kruskal-Wallis/Wilcoxon(*)*	*p-value*
Log(latency reach line)	0.61	0.737
Number of visits to model frog	221.5(*)	0.444
Number of visits to speaker	327(*)	0.077
Attack to the model frog (yes/no)	245.5(*)	0.846

Information on Bayesian models characteristics: For the models with the latency to reach the setup and the ratio (number of visits to the model/number of visits to the speaker) as response variables, we ran the models with 1,000,000 iterations, 10,000 burnin, and 100 thinning interval, with the following prior:

```
prior1 <- list(R = list(V = 1, nu = 0.002),
```

```
            G = list(G1 = list(V = 1, nu = 0.002), G2 = list(V = 1, nu = 0.002)))
```

For the models with the likelihood to attack the model frog and which stimuli was visited first as response variables, we ran the models with 2,000,000 iterations, 10,000 burnin, and 300 thinning interval, with the following prior:

```
prior2 <- list(R = list(V = 1, fix = 1),
            G = list(G1 = list(V = 1, nu = 0.002), G2 = list(V = 1, nu = 0.002)))
```

Table S1: Parameter estimates and test statistics for the Bayesian linear mixed models looking at the influence of the interactions between treatment and aggressiveness, and treatment and size on the behavioural variables measured during the bimodal test.

log(latency reach line)	*post.mean*	*l-95%*	*u-95%*	*eff.samp*	*pMCMC*
location	174.2	0	5.39	9900	
ID	0.15	0.01	0.3	9900	
intercept	6.64	2.85	10.25	9900	0.011
treatment 30	-0.98	-4.59	2.79	9329	0.595
treatment 60	-0.84	-4.52	3.01	9900	0.656
aggressiveness	-0.26	-0.55	0.02	9900	0.076
body size	-0.74	-1.93	0.32	9900	0.198
treatment 30 * aggressiveness	-0.04	-0.32	0.24	9900	0.791
treatment 60 * aggressiveness	-0.12	-0.41	0.15	9900	0.382
treatment 30 * body size	0.39	-0.87	1.66	9316	0.535
treatment 60 * body size	0.32	-1.07	1.49	9900	0.617
likelihood to attack the model frog	*post.mean*	*l-95%*	*u-95%*	*eff.samp*	*pMCMC*
location	6499	0	50.54	6634	
ID	6.4	0	20.09	2223	
intercept	20.73	-6.85	50.86	6634	0.114
treatment 30	-24.12	-56.52	5.87	5099	0.1
treatment 60	-26.81	-63.21	4.99	5143	0.091
aggressiveness	-1.27	-3.97	1.09	4107	0.269
body size	-6.54	-16.3	2.23	5529	0.113
treatment 30 * aggressiveness	0.8	-1.76	3.22	5427	0.53
treatment 60 * aggressiveness	0.44	-2.25	3.46	6634	0.773
treatment 30 * body size	7.78	-2.13	18.88	5200	0.117

treatment 60 * body size	7.93	-3.01	19.77	5323	0.146
first interaction	*post.mean*	*l-95%*	*u-95%*	*eff.samp*	*pMCMC*
location	199.3	0	25.47	6634	
ID	0.79	0	3.78	3406	
intercept	-3.85	-30.22	18.19	6634	0.817
treatment 60	12.27	-15.2	44.26	6364	0.429
aggressiveness	-1.01	-2.76	0.73	6371	0.225
body size	0.62	-6.73	9.23	6801	0.956
treatment 60 * aggressiveness	1.69	-0.58	3.97	6344	0.134
treatment 60 * body size	-3.02	-13.6	6.48	6390	0.591
ratio visits to the model/visits to the speaker	*post.mean*	*l-95%*	*u-95%*	*eff.samp*	*pMCMC*
location	50.48	0	3.71	9900	
ID	0.03	0	0.12	9371	
intercept	-1.04	-4.48	2.65	9900	0.564
treatment 60	-0.09	-4.53	4.04	9900	0.976
aggressiveness	0.06	-0.27	0.38	9900	0.672
body size	0.39	-0.72	1.51	9900	0.485
treatment 60 * aggressiveness	-0.17	-0.58	0.25	9900	0.407
treatment 60 * body size	-0.17	-1.62	1.3	9900	0.805
